# Mercury Exposure, Gene Expression, and Intelligence Quotient in Afro-Descendant Children from Two Colombian Regions

**DOI:** 10.3390/toxics13090786

**Published:** 2025-09-17

**Authors:** Javier Galvis-Ballesteros, Margareth Duran-Izquierdo, Juan Valdelamar-Villegas, Lucellys Sierra-Marquez, Jesus Olivero-Verbel

**Affiliations:** Environmental and Computational Chemistry Group, School of Pharmaceutical Sciences, Zaragocilla Campus, University of Cartagena, Cartagena 130015, Colombia; jgalvisb@unicartagena.edu.co (J.G.-B.); mdurani@unicartagena.edu.co (M.D.-I.); jvaldelamarv1@unicartagena.edu.co (J.V.-V.); lsierram@unicartagena.edu.co (L.S.-M.)

**Keywords:** oxidative stress, gene expression, gold mining, intelligence quotient

## Abstract

The impact of mercury (Hg) on biological systems is well documented; however, the long-term effects of low-level exposure in children remain unclear, particularly with respect to oxidative stress and cognitive outcomes. This study evaluated Hg exposure and its associations with the gene expression and intelligence quotient (IQ) in two Afro-descendant child populations in Colombia. Hair total mercury (T-Hg) was quantified in 163 children under 7 years old, along with their sociodemographic data. Significant differences (*p* < 0.05) were found in fish consumption and mean hair T-Hg concentrations between children from Mahates (2.66 ± 0.30 meals/week; 0.32 ± 0.03 µg/g) and Zanjón (1.24 ± 0.09 meals/week; 0.24 ± 0.01 µg/g). The gene expression analysis revealed higher SOD1 expression in Mahates. The mean IQ scores were higher in Zanjón (74.7) than those in Mahates (71.7). Overall, Spearman’s correlation analysis showed no significant associations (*p* > 0.05) between T-Hg and the measured variables. The principal component analysis (PCA) revealed a clear separation between populations: Mahates, associated with a higher mercury burden and the upregulation of stress-response genes, and Zanjón, characterized by a better cognitive performance and lower mercury exposure. These findings suggest that despite the low overall exposure and non-significant bivariate correlations, the communities displayed distinct profiles, highlighting the value of integrated molecular–cognitive biomonitoring and motivate longitudinal studies addressing co-exposures and socioeconomic confounding.

## 1. Introduction

Mercury is a global pollutant that poses significant public health concerns. Atmospheric deposition can easily reach ecosystems, thereby having a significant environmental impact [1]. Once converted into methylmercury (MeHg), it can bioaccumulate through the food chain, with contaminated fish being the primary source of exposure to biological systems through food consumption [2]. Numerous factors impact Hg exposure levels and concentrations in biological matrices. Artisanal and small-scale gold mining (ASGM) serves as the primary anthropogenic source of T-Hg in the environment [3,4]. Human exposure to Hg takes place through occupational or dietary exposure in several regions across Asia, Africa, and South America, including Colombia [5].

Populations living in regions affected by mining activities are at significant risk of experiencing the adverse effects of metal exposure, which can manifest in multiple organ systems, especially in vulnerable populations [6]. Examples of these health problems include neurological problems, motor and reproductive disorders, immunological conditions, cardiovascular and genetic abnormalities, and nephrotoxic and hepatotoxic problems [7]. In preschool children, whose physiological characteristics make them more susceptible to T-Hg exposure [8,9,10], effects of low-level Hg exposure on brain-derived neurotrophic factors have been reported [10].

The health impacts of T-Hg exposure are known; however, its molecular-level effects are yet to be determined [11]. One of the primary mechanisms activated during metal exposure is oxidative stress, which results from an imbalance between antioxidant and oxidant molecules in the cells [12,13]. This imbalance increases the amount of reactive oxygen species (ROS), leading to the formation of unstable molecules that have a strong binding capacity to biomolecules such as lipids, DNA, and proteins. Antioxidant enzymes, including catalase (CAT) and superoxide dismutase (SOD), and selenoenzymes like glutathione peroxidase (GPx) and thioredoxin reductase (Trx) safeguard the cells from the harmful effects of ROS production and have the capacity to prevent and reverse oxidative damage in a variety of tissues, particularly the liver and the brain [13].

In order to combat the toxic effects of heavy metals, organisms employ a defense mechanism involving metallothioneins (MTs) for detoxification. These metal-transporting proteins are characterized by their high cysteine content, which enables them to bind and sequester metals [14]. A study by Tejeda-Benitez et al. investigated the impact of trace elements in Magdalena River sediments on MT expression using an animal biological model. The findings revealed moderate T-Hg contamination along the river, with the highest levels concentrated at its mouth in the Colombian Caribbean. This study also observed a correlation between high T-Hg levels and elevated MT-2 gene expression, suggesting a biological response to the contamination. Similar patterns of contamination have been identified in the Cauca River basin, specifically in Zanjón Garrapatero Community Council (Zanjón), where the soil, water, fish and trees have been found to be contaminated with T-Hg [15].

The impacts of mercury on neurodevelopment in children are well documented. A systematic review in children from low- and middle-income countries (Bangladesh, n = 1; Brazil, n = 14; China, n = 3; Ecuador, n = 1) found that 8 of 17 studies reported an adverse association between mercury exposure and at least one neurodevelopmental domain [16].

Although some information is available on the relationship between low-level total mercury (T-Hg) exposure and the intelligence quotient (IQ) in children [9,12], no studies have been conducted along the Colombian coast characterizing the impacts of T-Hg contamination in children up to seven years old from Afro-descendant communities. The substantial T-Hg exposure observed in the study area, together with its known adverse health effects, provided the rationale for this investigation. This study evaluates the T-Hg exposure levels and their associated molecular and cognitive impacts in children from two Afro-descendant communities: Mahates, located along the Dique Channel, a tributary of the Magdalena River, and Zanjón, situated in an area influenced by the Cauca River.

## 2. Materials and Methods

### 2.1. The Study Area and Population

This study began with a meeting of community leaders, who were provided with various aspects of research information. Children up to 7 years old from two rural communities of Afro-descendant people were included in this study. The study was conducted from January to June of 2020. For all participants, informed consent was obtained from their parents. The first community, Remanso de Paz, has a population of 302 residents, 87 of whom are children under the age of 7. The community is located in the municipality of Mahates, which has a rural population of 26,075 residents, according to municipal sources (https://www.dane.gov.co/). Mahates comprises Afro-Colombian, Raizal, and Palenquero communities. It is located on the banks of the Dique Channel in the northern part of the Department of Bolívar, with coordinates of 10°14′14″ N and 75°11′19″ W.

The Dique Channel is a hydrological basin that facilitates fluvial transportation between the Magdalena River and the Caribbean Sea. It empties into the Bay of Cartagena, which houses the Port of Cartagena, the busiest container port in the Americas. The channel receives domestic, agricultural, and gold mining waste discharged from the central and northern regions of the country. The Dique and its marshes are the primary sources of water for agriculture and fishing in the region, representing the main economic activities [17].

**Figure 1:**
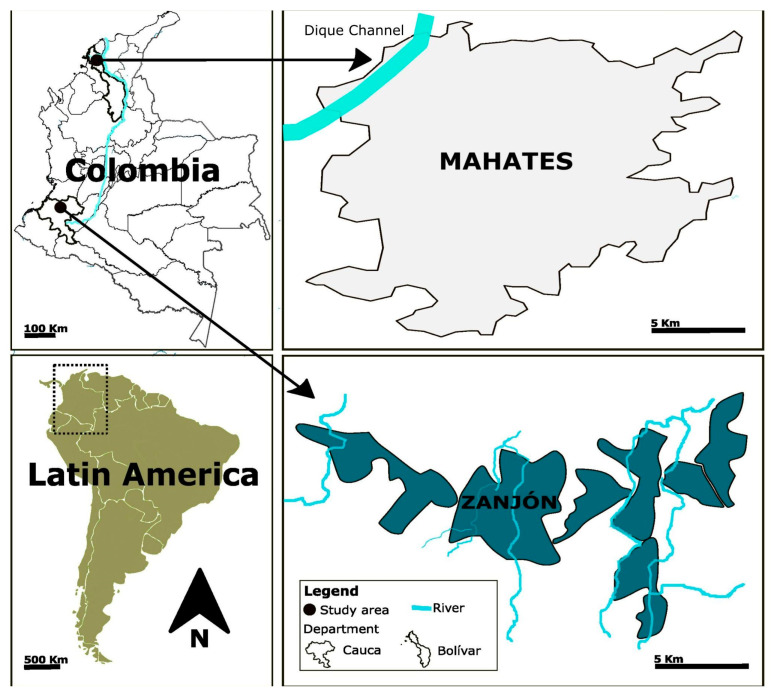
A location map of the study area.

The second community studied was Zanjón (Southwest Colombia), located in the municipality of Santander of Quilichao, the Department of Cauca (3°06′00″ N, 76°31′00″ W). It is home to 2536 inhabitants and encompasses an area of 5136 ha, which includes the communities of Ardovelas, Santa Lucia, El Palmar, Mazamorrero, Alto Palmar, Bajo San Francisco, and La Toma (see Figure 1). The territory has received different anthropogenic interventions, with gold mining being the one with the greatest environmental and social impacts [18].

### 2.2. The Questionnaire

The representatives of the selected participants completed a health questionnaire following the World Health Organization’s guidelines for identifying populations at risk of T-Hg exposure [19]. Individuals diagnosed with cognitive disabilities were excluded. In total, this study included 163 children. In this study, a total of 38 children from Mahates participated, representing 44% of the children under 7 years living in the Remanso de Paz community, which guaranteed high representativeness, despite the numerical disparity with the children evaluated in Zanjón.

### 2.3. Assessment of Hair T-Hg Concentrations

A total of 20 to 200 milligrams of hair samples was collected from the occipital region of the head using sterile stainless-steel scissors and then placed into paper envelopes at room temperature [5]. In the laboratory, the samples were cut into fragments measuring 1–2 mm before being washed with Milli-Q water (Millipore Sigma, Darmstadt, Germany), Triton X-100 (2%), and acetone. Subsequently, the samples were dried in an oven set to 40 °C [20] and preserved in microtubes within a desiccator until analysis.

The concentrations of T-Hg in the hair samples were quantified using an RA-915+ Zeeman Mercury Analyzer (Lumex, St. Peterburgo, Russia). The analysis was performed with the RP-915P software and the RP-91c pyrolysis unit (Lumex, St. Peterburgo, Russia). The protocol of the United States Environmental Protection Agency was followed [21], and calibration curves were generated for all cases using certified the reference materials of human hair (CRMs). The calibration curves were deemed optimal when the R^2^ value was ≥0.99. The analytical precision was determined using IAEA-086 (International Atomic Energy Agency, Analytical Quality Control Services, Viena, Austria), a material sourced from the International Atomic Energy Agency in Vienna, Austria, with a concentration of 0.573 µg/g. The precision was calculated as the relative standard deviation for duplicate determinations, which is typically less than 10%. The instrumental limit of detection of 0.001 µg/g was calculated using the standard deviation for blanks multiplied by three.

### 2.4. The Gene Expression Analysis

For the gene expression assays, blood samples were collected through venipuncture using a BD Vacutainer and vacuum tube needles. Standard aseptic techniques were employed. Gene expression studies were conducted to determine the effects of T-Hg exposure on the cells. To isolate total RNA from the whole blood samples, the Tempus™ Blood RNA Tube (Applied Biosystems, Foster City, CA, USA) was employed. Each tube in the Tempus Blood RNA Kit contained 6 mL of Stabilizing Reagent and allowed up to 3 mL of blood to be collected. In accordance with the manufacturer’s instructions, the samples for mRNA analysis were stored at −80 °C as soon as they arrived in the laboratory [22].

Spectrophotometry using a NanoDrop 2000 (Thermo Scientific, Wilmington, NC, USA) was employed to measure RNA concentrations, and the A260/A280 ratio was used to assess purity. DNA synthesis was conducted employing the high-capacity cDNA reverse transcription kit from Applied Biosystems in Foster City, CA, USA. RT-PCR was conducted utilizing the SYBR Green qPCR Master Mix from Thermo Scientific in Wilmington, NC, USA, on a Step One Plus thermal cycler from Applied Biosystems. The conditions for RT-PCR followed the manufacturer’s recommendations. Three genes related to metal exposure and oxidative stress were examined: metallothionein 1A (MT1A), 1K (MT1K or MT1M), and superoxide dismutase 1 (SOD1). The comparative CT method (ΔCT) was employed to evaluate these genes, using the geometric mean of glyceraldehyde-3-phosphate dehydrogenase (GAPDH) and beta-2-microglobulin (β2M) as reference genes [23]. All technical term abbreviations are explained in the text. Negative controls without cDNA were also used during the experiments.

### 2.5. The Cognitive Assessment

The previously validated Kaufman IQ test was employed in Colombia [22] to obtain the total IQ scores and two ability scores, specifically the vocabulary scale (VoS) and the matrices scale (MaS), for children. The evaluations were conducted by an experienced psychologist in separate, one-on-one rooms, in areas where the noise was minimal. Each test lasted between 25 and 60 min. Scores below 70 are considered to be very low, while scores above 129 are very high.

### 2.6. The Statistical Analysis

The data are presented as the mean ± standard error. For each variable, a frequency distribution, measures of central tendency, and variability were assessed. Prior to the analysis, normality tests were conducted using the Shapiro–Wilk and Bartlett tests. Mean comparisons were conducted using either the *T*-test or the Mann–Whitney *U* test, depending on whether the data were parametric or nonparametric. The associations between quantitative variables were analyzed using Spearman’s correlation. Statistical significance was set at *p* < 0.05. Furthermore, a multivariate analysis was conducted using a principal component analysis (PCA) to examine the spatial patterns between communities, Hg concentrations, and gene expression and IQ. The statistical analysis was conducted using GraphPad Prism 6.0 and IBM SPSS Statistics 25 software. Prior to their application, both the questionnaire and the cognitive test were validated for internal consistency using Cronbach’s alpha, obtaining coefficients above the acceptable threshold (α > 0.70).

## 3. Results

### 3.1. Sociodemographic Data

The general characteristics of the study population are presented in Table 1. The sample consisted of 163 children, with 38 from Mahates and 125 from Zanjón. The children were aged between 0 and 7 years. In the Mahates population, there was equal participation of boys and girls. In contrast, the Zanjón population exhibited a higher proportion of female participants (55.2%). No significant differences were observed in gender or exposure to tobacco distribution between the two populations (*p* > 0.05). The participants were distributed unevenly across the study sites with regard to age (*p* < 0.05).

All participants were classified within the lowest socioeconomic stratum of the six available categories. An analysis of the economic situation of the households in Mahates revealed an average income of COP 526.191 ± 276.988. In the community of Zanjón specifically, the average income was COP 519.323 ± 334.026, with no significant difference between this figure and the overall average (*p* > 0.05). Regarding the diversity of foods consumed, different patterns were identified among the populations. Children in Mahates reported lower fruit and vegetable consumption (89.5%) than those in Zanjón (94.4%). Notably, the average weekly fish consumption was significantly higher in Mahates (2.66 ± 0.30 meals/week) than that in Zanjón (1.24 ± 0.09 meals/week), with a statistically significant difference (*p* < 0.05).

Zanjón shows a significantly higher rate of high school attainment among parents/guardians at 74%, nearly double that in Mahates at 37% (*p* < 0.05). This highlights a notable educational gap within the broader community. Conversely, pesticide use in households is more prevalent in Mahates, with 71% of parents/guardians reporting their use, compared to 45% in Zanjón (*p* < 0.05).

### 3.2. T-Hg Hair Concentrations

**Figure 2:**
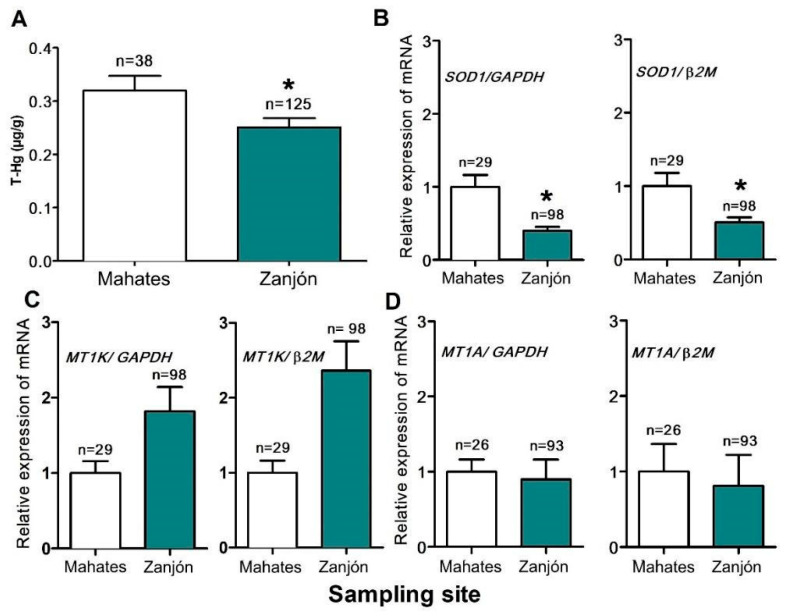
T-Hg concentrations in hair (**A**) and gene expression in children from Mahates and Zanjón. The SOD1 (**B**), MT1K/1M (**C**), and MT1A (**D**) genes were subjected to analysis. The changes in gene expression were quantified using *GAPDH* and β*2M* as reference genes. * A statistically significant mean difference was observed when the Zanjón data was compared to that for the Mahates group (*p* < 0.05).

The mean hair T-Hg concentrations in the children from Mahates and Zanjón are shown in Figure 2A, with the medians and ranges provided in Table S1. The hair T-Hg concentrations for the children from Mahates were found to be significantly higher than those in the children from Zanjón (*p* < 0.05) (see Figure 2A). The mean concentrations for Mahates were 0.32 ± 0.03 µg/g (range: 0.04–0.83 µg/g), while those for Zanjón were 0.24 ± 0.01 µg/g (range: 0.05–1.50 µg/g). The group means were below 1 µg/g; however, individual values in Zanjón reached 1.50 µg/g, exceeding the international guideline (1 μg/g).

### 3.3. The Gene Expression Results

In relation to the findings, it was observed that the SOD1 gene expression was higher in the children from Mahates (*p* < 0.05) compared to that in those from Zanjón. This pattern was similar to that for MT1A gene expression; however, there were no significant differences between the two groups in MT1A gene expression. Conversely, the mean MT1K/1M gene expression in the individuals assessed in the Zanjón group was higher than that observed in the Mahates group, although no significant intergroup differences were observed between these groups (Figure 2B–D).

### 3.4. The Cognitive Test Results

**Figure 3:**
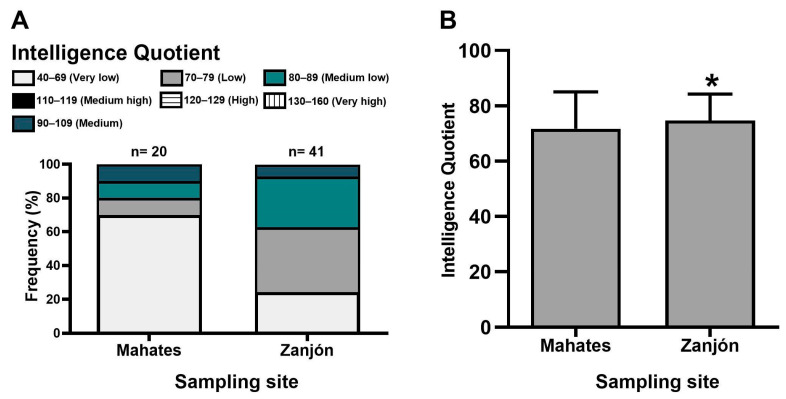
IQ evaluated in children residing in the Mahates and Zanjón communities. Relative frequency distribution of IQ (**A**); mean ± SEM (**B**). * A statistically significant mean difference was observed when the Zanjón data was compared to that for the Mahates group (*p* < 0.05).

As the Kaufman test has been validated exclusively for use in individuals aged four and above, we administered it to 20 children from Mahates and 41 from Zanjón. The IQ scores of the children from Mahates and Zanjón are presented in Figure 3. It is noteworthy that 70% of the children in Mahates exhibited an IQ within the minimum range (very low), while 24.39% of the children assessed in Zanjón also fell within this range (Figure 3A).

Notably, only 10% of the children in Mahates and 7.32% in Zanjón scored within the medium–high IQ range, and no individuals with high or very high levels were identified in either community. Furthermore, although both communities demonstrated average scores on the second scale (low), the children from Mahates exhibited a significantly lower mean IQ score (71.7) compared to that for those from the Zanjón community (74.7) (*p* < 0.05).

### 3.5. Relationships Between the Concentration of T-Hg in the Hair, Gene Expression, and IQ

The correlation analysis in Table 2 shows that overall, none of the analyzed variables (age, fish intake, SOD1, GAPDH, MT1K/1M, MT1A, IQ, VoS, MaS) were significantly correlated (*p* > 0.05) with total mercury content (T-Hg) in the children from Mahates or Zanjón. The Spearman’s correlation coefficients (*ρ*) ranged from −0.017 (T-Hg vs. SOD1/β2M) to 0.31 (T-Hg vs. MT1A/β2M) in Mahates and from −0.135 (T-Hg vs. SOD1/β2M) to 0.051 (T-Hg vs. MaS) in Zanjón.

**Figure 4:**
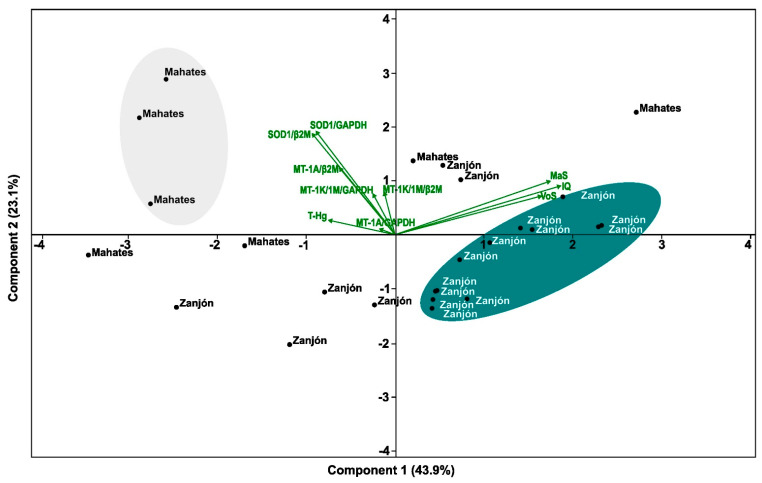
Diagram of principal component analysis for selected variables.

A principal component analysis was used to assess the multivariate relationships among the study variables (Figure 4). The PCA clearly separates the two populations. Individuals from Mahates cluster on the negative side of Component 1 (43.9%), associated with higher total mercury (T-Hg) levels and increased expression of metallothionein (MT) and SOD1 genes, suggesting stronger responses to metal-induced oxidative stress. In contrast, individuals from Zanjón cluster on the positive side of Component 1, where the cognitive variables (IQ, vocabulary score, matrices) load positively, indicating a better neurocognitive performance with a lower mercury burden. Component 2 captures additional variability (23.1%) but contributes less to the population separation. Overall, the analysis highlights distinct environmental and biological profiles: Mahates shows higher mercury exposure and adaptive gene activation, while Zanjón demonstrates better cognitive outcomes.

## 4. Discussion

This study is pioneering in its assessment of T-Hg exposure, cognitive development, and gene expression in Afro-descendant children from Mahates and Zanjón, thus distinguishing it from previous research, which was primarily focused on mining areas in Colombia. However, the T-Hg concentrations observed in the hair were notably lower than those reported for other Colombian child populations [24,25], as well as in studies from Brazil [26], Mexico [27], and Asia [28,29,30,31], among others (Table S2).

While the T-Hg levels in the children in this study were lower than those in the Faroe Islands (a seafood-dependent country) [32], they were notably higher than those observed in children from the U.S. from the NHANES 1999–2000 survey, which reported a geometric mean of 0.12 µg/g in the hair for children aged 1–5 years [33].

Overall, the mean fish consumption across both Colombian communities was low (1.5 ± 0.06 meals/week), as were the T-Hg concentrations (0.28 ± 0.01 µg/g). However, children from Mahates ate fish more frequently (2.66 ± 0.30 meals/week) and had higher T-Hg levels than those in children from Zanjón. The children from Zanjón also showed higher fruit and vegetable consumption (94.4%) compared to that in Mahates (89.5%). This suggests that children in Mahates might be more reliant on fish, potentially leading to less dietary diversity and a greater risk of T-Hg exposure. Future research should measure T-Hg in fruits and vegetables from both communities to confirm this.

The lower T-Hg levels in this study likely stem from dietary differences and less frequent consumption of fish compared to that in other Colombian and global populations. The primary source of exposure to T-Hg is contaminated fish [20]. Increased awareness of T-Hg’s effects, supported by local research [24,34], may contribute to this. However, Colombia remains the top per capita T-Hg emitter globally [35], necessitating continued monitoring of children’s exposure in the region as global efforts focus on pollution reductions [36]. Vulnerable communities face heightened T-Hg exposure. A recent study in the Colombian Amazon, aligning with prior research [37], connected higher T-Hg levels in the hair to fish consumption. Specifically, individuals that ate fish 1–14 times weekly had significantly lower T-Hg levels (18.6 ± 1.6 µg/g) than those consuming it more than 14 times weekly (27.1 ± 1.6 µg/g) [38].

While our study found low T-Hg levels in these children, further analysis is needed to confirm its conclusions. Other factors, such as income and education levels, are known to influence IQ in communities near mining areas [29]. This study also revealed significant differences in pesticide use by the parents in Mahates, a factor that could significantly impact neurodevelopment.

Although Mahates and Zanjón differed in their fish consumption, hair T-Hg levels, SOD1 expression, and mean IQ scores, no significant associations were detected between T-Hg and the variables evaluated. These findings contrast with those of previous studies [31,37], where fish consumption was identified as a common source of mercury exposure. Nevertheless, the PCA results revealed distinct community profiles: Mahates, located next to the Canal del Dique, clustered toward relatively greater mercury exposure and SOD1 expression, while Zanjón was characterized by a comparatively better cognitive performance. The Canal del Dique, which connects the Magdalena River with the Caribbean Sea, has been reported to carry elevated T-Hg levels in its sediments due to upstream gold mining activities [14]. As this channel remains an important source of fish and agricultural products for Mahates, the potential for bioaccumulation in local species cannot be disregarded. In contrast, decades of mining activity in the Cauca River basin [18] have reduced the local availability of fish in Zanjón, leading to the diversification of protein sources and reliance on fish imports of an uncertain origin.

While environmental pollutants’ impact on oxidative stress is well researched [39], the effects of low-level toxic metal exposure, specifically T-Hg, on children, especially concerning gene expression, are poorly understood [40]. In our study, SOD1 was the only gene that showed significantly higher expression in Mahates, aligning with its role as a key antioxidant enzyme induced by metal exposure [41,42]. By contrast, MT1A and MT1K/1M did not differ significantly between groups, suggesting that their expression patterns may be influenced by additional factors beyond mercury exposure [43,44,45,46,47]. This emphasizes that SOD1 may be a more sensitive biomarker of low-level Hg exposure in children than MTs in this context.

Metallothioneins are vital for metal transport and detoxification and are key indicators of the body’s antioxidant response to contaminants. While MT1A expression has been linked to T-Hg exposure and cognitive decline in adults [43] and oxidative stress due to T-Hg has been studied in adult consumers of fish [44], research on children remains limited [9,12]. For example, a study in China found that children (8–10 years) exposed to T-Hg through contaminated rice had hair T-Hg levels 65.6% above the recommended limits, with those exceeding 1 µg/g being 1.58 times more likely to have an IQ below 80 (the intellectual disability threshold) [29].

However, previous studies have described that MT1A, compared to SOD1, has low specificity; its expression is influenced by various pollutants, including cadmium [39], arsenic [45], copper, and PM10 [46], as well as conditions like neoplasms [47] and cancer. Furthermore, elevated T-Hg exposure is linked to lower IQ scores in children as young as 60 months [29], with high T-Hg levels, particularly due to late prenatal exposure, correlating with cognitive deficits [48].

While some studies [49] found no direct link between prenatal T-Hg and intellect, recent Colombian research shows a decline in IQ in children (9–16 years old) from Caribbean mining areas due to T-Hg and arsenic exposure [28]. Despite limited evidence on low-level exposure for prenatal T-Hg in particular, neurological effects have been mainly associated with exposure to other metals such as lead [50], aligning with the understanding that the developing brain is highly vulnerable to neurotoxicants like T-Hg, risking permanent damage [51]. T-Hg’s cognitive impact stems from its affinity for neurological tissue, accumulating in the astrocytes and interfering with glutamate and aspartate reuptake, leading to neuronal toxicity [29]. This poses a significant public health risk due to irreversible effects in children, even at low exposure levels [29,52]. Although both communities showed generally low IQ scores, children from Zanjón performed slightly but significantly better than those from Mahates. This modest difference (74.7 vs. 71.7) is consistent with the PCA results, where the cognitive variables loaded positively onto the Zanjón cluster. These findings indicate that while a low cognitive performance was prevalent across both populations, the relative advantage observed in Zanjón deserves further exploration.

This study, despite its innovative assessment of the T-Hg exposure, cognitive development, and gene expression in Afro-descendant children from Mahates and Zanjón, faces several limitations, requiring cautious interpretation. A key constraint is that the T-Hg concentrations observed in the hair were lower than those reported in other Colombian and global populations of children, indicating that these findings may not be generalizable to communities with higher mercury exposure. Future research is strongly recommended to thoroughly investigate additional sociodemographic factors, such as parental education levels, and other pollutant exposures like pesticides (which significantly differed between the studied communities), as these could significantly impact neurodevelopment and introduce biases not fully explored in this study.

Consequently, it is imperative that future research endeavors focus on the collection of comprehensive data on these variables. This study aligns with international guidelines, including those from the European Commission, which emphasize testing for T-Hg health effects, particularly in vulnerable populations. It also follows the World Health Organization’s recommendations for evaluating groups exposed to varying T-Hg levels [53]. These guidelines generally promote epidemiological studies to clarify the metal’s health impacts, a goal met by this work, which identified low exposure levels in highly vulnerable communities.

## 5. Conclusions

This study demonstrates that Afro-descendant children from Mahates and Zanjón exhibit distinct multivariate profiles, with Mahates clustering toward relatively greater mercury exposure and SOD1 upregulation and Zanjón showing comparatively better cognitive outcomes. Although the overall mercury concentrations were low and no significant correlations were found between Hg levels, gene expression, and cognitive variables, the PCA highlighted clear differences between the two populations. These findings suggest that even in the absence of direct associations in bivariate analyses, multivariate approaches can reveal differentiated community profiles. Future longitudinal studies are required to clarify these patterns and assess the potential long-term effects of low-level mercury exposure on gene expression and neurodevelopment.

## Figures and Tables

**Table 1:** Sociodemographic characteristics and dietary and health habits of children in Mahates and Zanjón.

Variable	Categories	Study Area		Statistic	*p*-Value
		Mahates *n* (%)	Zanjón *n* (%)		
Age (years)	≤4 years	18 (47.4)	84 (67.2)	*X*^2^ = 4.89	0.023 *
	>4 years	20 (52.6)	41 (32.8)		
Sex	Girl	19 (50.0)	69 (55.2)	*X*^2^ = 0.32	0.352
	Boy	19 (50.0)	56 (44.8)		
Educational level ^a^	None	2 (5.0)	0 (0.0)	*X*^2^ = 29.5	0.006 *
	Elementary school	8 (21.0)	17 (14.0)		
	High school	14 (37.0)	93 (74.0)		
	Technical	11 (29.0)	11 (9.0)		
	Technologist	1 (3.0)	3 (2.0)		
	College	0 (0.0)	1 (1.0)		
	DK/NA ^b^	2 (5.0)	0 (0.0)		
	0	1 (2.6)	32 (25.6)	*X*^2^ = 33.5	0.001 *
Fish intake (meals/week)	1–2	21 (55.3)	85 (68.0)		
	≥3	16 (42.1)	8 (6.4)		
	Mean ± SEM	2.66 ± 0.30	1.24 ± 0.09	*U* = 1154	0.001 *
Exposure to tobacco ^i^	Yes	8 (21.1)	14 (11.2)	*X*^2^ = 2.42	0.102
	No	30 (78.9)	111 (88.8)		
Pesticide use ^a^	Yes	27 (71.0)	56 (45.0)	*X*^2^ = 12.3	0.006 *
	No	10 (26.0)	68 (54.0)		
	DK/NA ^b^	1 (3.0)	1 (1.0)		

^i^ Passive exposure to tobacco smoke; *X*^2^ = Chi-square; *U =* Mann–Whitney *U* test. ^a^ Parent/guardian. ^b^ Do Not Know/No Answer. * A statistically significant difference between groups (*p* < 0.05).

**Table 2:** Spearman’s correlations between T-Hg concentrations in hair and age, fish intake, IQ, and molecular variables in children from Mahates and Zanjón.

Variables	Study Area			
	Mahates		Zanjón	
	Spearman Correlation	*p*-Value	Spearman Correlation	*p*-Value
Age	0.164	0.394	−0.015	0.890
Fish intake ^i^	0.113	0.559	0.044	0.695
*Molecular*				
SOD1/β2M	−0.017	0.932	−0.135	0.225
SOD1/GAPDH	−0.077	0.691	−0.090	0.421
MT1K/1M/β2M	0.061	0.760	−0.111	0.318
MT1K/1M/GAPDH	0.166	0.390	−0.053	0.634
MT1A/β2M	0.313	0.119	−0.131	0.257
MT1A/GAPDH	0.310	0.123	−0.130	0.260
*IQ*				
Score	−0.321	0.482	0.032	0.886
Vocabulary scale (VoS)	−0.179	0.702	0.004	0.987
Matrices scale (MaS)	−0.393	0.383	0.051	0.817

^i^, meals/week.

## Data Availability

The data cannot be disclosed due to confidentiality reasons.

## Supplementary Materials

The following supporting information can be downloaded at https://www.mdpi.com/article/10.3390/toxics13090786/s1, Table S1: The medians and ranges of hair T-Hg concentrations (µg/g) in children from the studied communities. Table S2: Summary of studies reporting Hg concentrations in hair (µg/g) of children from different places.
