# Supplementary material for: Mercury Exposure, Gene Expression, and Intelligence Quotient in Afro-Descendant Children from Two Colombian Regions
Source: Toxics. 2025 Sep 17;13(9):786. doi: 10.3390/toxics13090786 (PMC12474032; doi:10.3390/toxics13090786)
Supplement: Supplementary file 1 [file toxics-13-00786-s001.zip › toxics-3720854-supplementary.pdf]

Toxics

Mercury Exposure, Gene Expression and Intelligence Quotient in Afro-Descendant Children from Two Colombian Regions

Table S1. Median, and range of children hair T-Hg concentrations (µg/g) in studied communities.

Study area	Median	Range (Min–Max)
Mahates	0.29	0.04-0.83
Zanjón	0.22	0.05-1.49

Table S2. Summary of studies reporting Hg concentrations in hair (µg/g) of children from different places.

Reference	Country	[Hg]	Age	N
This study	Colombia	0.28	0-7 years	163
De la Ossa et al. [24]	Colombia	1.76	9-16 years	70
Marrugo-Negrete et al. [25][a]	Colombia	0.40-24.56	2-15 years	24
Santos-Lima et al.[26]	Brazil	2.05	6-14 years	263
Basu et al. [27]	Mexico	0.56	10.3 years	825
McDowell et al. [33]	USA	0.22	1-5 years	838
Feng et al. [29]	China	1.53	8-10 years	314
Kusanagi et al. [31]	Japan	0.87	3–6 years	118
Kim et al. [30]	Korea	0.74	1–186 months	112
Gustin et al. [28][b]	Bangladesh	0.67	10 years	1434
Budtz-Jørgensen et al. [32]	Islas Feroe	0.60	7-14 years	903

[a] Range; [b] Median, and the rest is mean value;
[Hg] = Hg concentration.
